# Artificial data in sports forecasting: a simulation framework for analysing predictive models in sports

**DOI:** 10.1007/s10257-022-00560-9

**Published:** 2022-06-15

**Authors:** Marc Garnica-Caparrós, Daniel Memmert, Fabian Wunderlich

**Affiliations:** Institute of Exercise Training and Sport Informatics, German Sport University Cologne, Am SportPark Müngersdorf 6, 50933 Cologne, Germany

**Keywords:** Simulation, Knowledge modelling, Information systems frameworks, Sports forecasting, Artificial data generation

## Abstract

Far-reaching decisions in organizations often rely on sophisticated methods of data analysis. However, data availability is not always given in complex real-world systems, and even available data may not fully reflect all the underlying processes. In these cases, artificial data can help shed light on pitfalls in decision making, and gain insights on optimized methods. The present paper uses the example of forecasts targeting the outcomes of sports events, representing a domain where despite the increasing complexity and coverage of models, the proposed methods may fail to identify the main sources of inaccuracy. While the actual outcome of the events provides a basis for validation, it remains unknown whether inaccurate forecasts source from misestimating the strength of each competitor, inaccurate forecasting methods or just from inherently random processes. To untangle this paradigm, the present paper proposes the design of a comprehensive simulation framework that models the sports forecasting process while having full control of all the underlying unknowns. A generalized model of the sports forecasting process is presented as the conceptual basis of the system and is supported by the main challenges of real-world data applications. The framework aims to provide a better understanding of rating procedures and forecasting techniques that will boost new developments and serve as a robust validation system accounting for the predictive quality of forecasts. As a proof of concept, a full data generation is showcased together with the main analytical advantages of using artificial data.

## Introduction

Forecasting future events is a challenging task that requires a high level of domain-specific understanding of the underlying processes, high quality and availability of data, as well as detailed statistical modelling. In real-world situations, uncertainty governs all different steps of decision making, and one of the main tasks of accurate forecasting is to embrace this uncertainty to support decisions. The increased relevance and complexity has led to forecasting becoming a highly interdisciplinary research topic that deals with areas as diverse as economics (Timmermann 2000), politics (Wolfers and Leigh 2002), energy supply (Hong et al. 2016), weather (Taylor and Buizza 2004), climate (Green et al. 2009), criminality (Gorr et al. 2003), or demography (Booth 2006). The present paper is focused on another well-established and widely studied forecasting domain, which addresses the outcomes of sports events (McHale and Swartz 2019; Stekler et al. 2010; Wunderlich and Memmert 2020b).

Three particular aspects drive the relevance of sports forecasting. First, the large and growing sports betting market (Nederlandse Online Gambling Associatie 2015) represents a real-world economic application, where wrong decisions on forecasting models might lead to negative financial consequences for bookmakers (Forrest et al. 2005) and economists find a viable data environment to investigate market efficiency (Angelini and Angelis 2019). Second, the great public interest in sports and broad media coverage of events supports a high availability of data, which makes it possible to assess decent sports-related datasets (Baker and McHale 2013; Kovalchik 2016; Štrumbelj and Šikonja 2010). Third, predictive models in sport can also contribute to answering sports science questions by providing a better understanding of the characteristics of the sports (Heuer and Rubner 2009; Štrumbelj and Vračar 2012; Wunderlich and Memmert 2018). This paper introduces the use of information technology in the sports forecasting domain from a foundational perspective, provides an abstraction of the processes and information involved and proposes the modelling, generation, and use of artificial data to study and improve forecasting scenarios.

Despite the fact that the existing knowledge in sports forecasting contains a plethora of statistical models (Wunderlich and Memmert 2020b) as well as an increasingly number of advanced data mining methods (Horvat and Job 2020; Lessmann et al. 2010), the present paper attempts to extent this body of research by critically examining each of the components participating in a very heterogenous information system. As reported by Venable et al. (2016), the formal description of the processes involved can boost research communication between practitioners as well as drive the scope of new developments. Moreover, the design and granular investigation of such a generic model of sports forecasting enables an integral simulation of the system. Other relevant fields of forecasting have robust integrations with simulation and modelling research such as weather forecasting (Wilks and Wilby 1999). However, even though the simulation and statistical modelling of data have been used to predict and validate sports outcomes, for instance, modelling competition structures in basketball (Saá Guerra et al. 2012) or simulating full tournaments result in tennis (Clarke and Dyte 2000) and football (Leitner et al. 2010), to the best of our knowledge, no prior research has presented a simulation-based approach accounting for all relevant steps of sports forecasting and evaluation of forecasts in sports.

The beneficial aspects of modelling and simulation of any environment subject to analysis are well established. From a data collection point of view, real-world datasets are difficult to use for inference learning and reasoning due to the amount of information and factors involved (Lin et al. 2014). Sometimes it is difficult to decouple noise from general-purpose collected datasets, and the generation of real data is usually restricted to time. The ability to control and understand artificial data’s inherent relationships is a major advantage that creates interest in the scientific community. Moreover, in certain topics, artificial data can be created faster and in larger quantities than real-world data is accessible. From a methodological point of view, numerous success cases have been highlighted in the conjunction of simulation applications in information systems and real-world applications, for instance, in healthcare (Jahangirian et al. 2012; Zhang 2018) or manufacturing (Mourtzis et al. 2014). Generating artificial data is used for simulation purposes and data sharing, benchmarking and testing software systems (Misra 2015).

The contributions of the present paper are the following: The process involved in sports forecasting is examined in detail from the existing literature, and a generalized definition is presented to include all sports competitions with a format of pairwise comparisons. Moreover, following the described process, a simulation framework that can be used to create and analyze artificial sports data is introduced. The instantiated framework constitutes a system able to represent all relevant aspects of forecasting in sports, which are timely event schedules (graphs) and event outcomes, the estimation of team or player strengths (ratings), the prediction of probabilities for various outcomes (forecasting) and the consideration of bookmakers (betting odds) and betting strategies to measure model profitability. The two-fold contribution of this research is effectively evaluated by their fit and integration in the sports forecasting environment (Hevner et al. 2004). The generalized process model is presented in concordance with the existent practices and exposes the challenges of current practitioners with real-data scenarios. The framework uses the presented model to structure and theoretically eliminate the exposed challenges. In conjunction, a detailed proof of concept is presented to showcase how the framework can be used to theoretically analyze sports forecasting methods and gain an improved understanding of sports forecasting processes.

## A generalized process of sports forecasting and current challenges in real-world data applications

The idea to establish a framework for investigating sports forecasting processes by means of artificial data is based on two prerequisites. First, the existence of a generalizable forecasting process in the state-of-the-art sports forecasting literature. Second, the existence of specific challenges when applying this process to real-world datasets, which can be overcome by using artificial data. Subsequently, a forecasting process is presented, that includes four major steps, is generalizable to a wide range of sports and has been widely deployed in real-world scenarios. Moreover, in each step, challenges arising from the characteristics of real-world datasets are explained. These challenges mainly stem from the fact that real-world data is limited in size, cannot be deliberately controlled and the true underlying processes of data generation are not directly observable. All of these drawbacks can be tackled by the use of artificial data generation.

The process is focused on competitions consisting of pair confrontations according to a prearranged competition schedule. This excludes sports where more than two actors compete at the same time in a joint competition to determine the winner (e.g. horse racing, golf, motorsports, ski racing, athletics, etc.) but includes a wide range of team sports (e.g. football (Constantinou et al. 2012), basketball (Manner 2016), American football (Baker and McHale 2013), cricket (Asif and McHale 2016), ice hockey (Marek et al. 2014), baseball (Soto Valero 2016), handball (Groll et al. 2020), etc.) and individual sports (e.g. chess (Glickman and Jones 1999), tennis (Kovalchik 2016), table tennis (Lai et al. 2018), darts (Liebscher and Kirschstein 2017), etc.) that represent common applications of sports forecasting.

The generality and cross-thematic approach of the process makes it impossible to present a full and comprehensive literature review including all relevant approaches and methods in sports forecasting across all sports. For a more detailed overview on the sports forecasting literature, we thus refer to the reviews of Wunderlich and Memmert (2020b) and Horvat and Job (2020), as well as to two special issues related to sports forecasting in the International Journal of Forecasting (see McHale and Swartz 2019, Vaughan Williams and Stekler 2010). Sports forecasting use cases are inherently a data collection and analysis process. The initial step involves the creation and configuration of the sports competitions (Step 1). That is, sports competitions are formed in a natural basis, they involve teams or players and a certain competition schedule. This schedule governs the evolution of confrontations and structure of the competition that will finally conclude with a set of conclusions premises (i.e., winner of the competition). In despite of other analytical scenarios, sports forecasting differentiates clearly between the evaluation of the competitors (Step 2) and the prediction of a certain match (Step 3). Finally, the predictive quality is evaluated (Step 4), once the actual results of the confrontations are known.

Table 1 summarizes the four steps. Each is discussed in detail below exposing the current limitation and challenges of real-world data scenarios. In the following section, each of the steps is integrated and formulated into the artificial data framework to cope with the exposed challenges.

**Table 1:** The sports forecasting process

Step	Description
Step 1: The competition network	The sports forecasting process is based on an existing scheduling of confrontations between competitors referred to as *network*. The network dictates how the competitors are going to face each other and how the competition evolves over time
Step 2: The rating procedure	The strength of each competitor is estimated by means of one or several quantitative measure(s)
Step 3: The forecasting method	Competitor confrontations and their potential outcomes are modelled by a combination of systematic and unsystematic effects including the fore-mentioned competitors’ ratings
Step 4: The model validation	Once the result of a confrontation is known, the forecast can be evaluated by two principles: The correctness or closeness of the predicted result to the actual result (*forecast accuracy*) and the economic return of a certain prediction (*forecast profitability*)

*Step 1: The competition network*

Sports data is created naturally by highly active sports disciplines, involving teams or players competing regularly in several leagues or tournaments. Limiting the analysis to sports with pairwise comparisons enables the schedule of play to be represented as a temporal network (Newman 2010), where competitors represent vertices and matches represent edges. The competition network can be characterized as the data basis for the forecasting process. In real-world scenarios, the forecaster does not have any direct influence on its creation. The network characteristics depend on the sport and competition. For instance, in a national European football league, teams play each other twice. The winner of the competition is the team with the best overall performance.

**Fig. 1:**
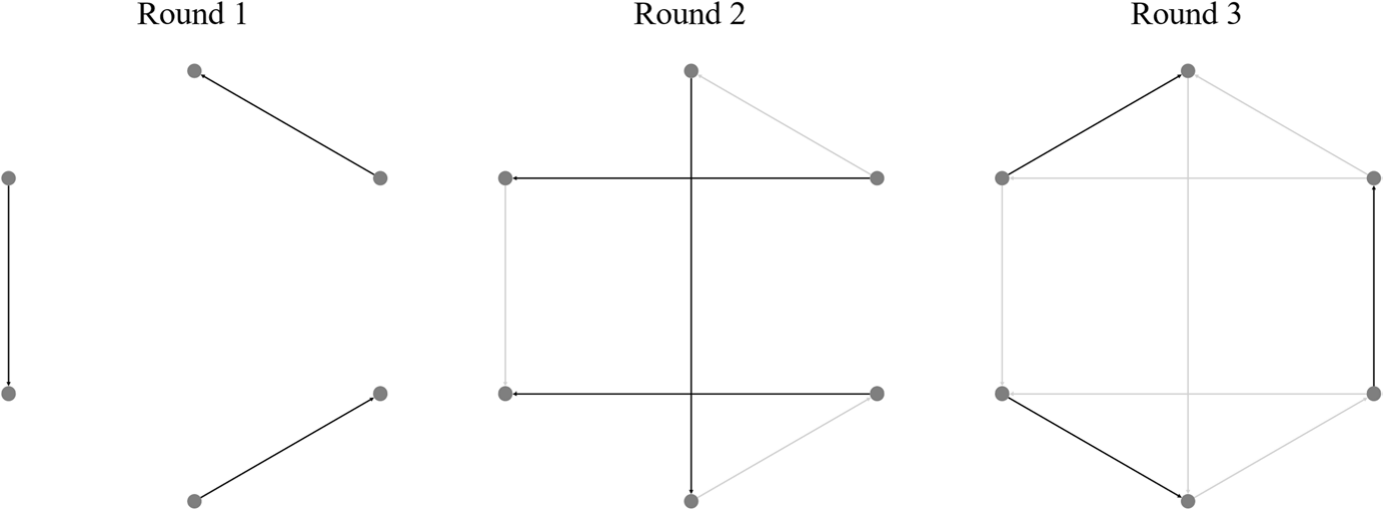
An example of a competition network and its temporality. Only a certain number of edges is activated on each round, colored as black. In addition, edges with known results, colored with light grey, are gradually being generated

Figure 1 illustrates the composition of a competition network and its inherent temporality. The nodes of this network refer to entities competing in a sports event, namely teams in sports like European football or basketball and individual players in disciplines such as tennis or chess. This network structure is highly flexible and reflects sport dynamics and organization. In some tournaments, edges can be repeated, while the comparison of two actors is unique on other occasions. If the sport requires, the edge direction can serve as a mapping to determine home and away teams on an event (Bang-Jensen and Gutin 2009). In elimination tournaments, the result of a single pairwise competition can change the following events, while in tournaments such as national leagues, the schedule remains predefined. Examples from the forecasting literature that explicitly discuss and illustrate the network structure of competitions can be found in the work of Lai et al. (2018). They model more than 700.000 Italian table tennis matches as a network, Wunderlich and Memmert (2018), discussing the asymmetry of within-league comparisons in national leagues and cross-league comparisons by means of international club competitions in football, or Juyong Park and M E J Newman (2005) illustrating the network of college American football matches with its conference system. The network itself can be accompanied with a lot of relevant data, such as match outcomes, betting odds, or additional player- or team-specific statistics. In most forecasting research, data is not discussed explicitly as a network; however, as long as targeting at the pairwise comparison, data will in every case be representable by this network structure.

A significant challenge in real-world applications is that data can be limited with regard to several aspects such as size, consistency and format rules of sports. Sample size, in general, is not the predominant challenge in sports, particularly compared to other forecasting domains. While data acquisition in economic or social forecasting might be complicated by company secrets or data protection, the outcomes of sports events and the betting odds from the betting market are published in the public domain and freely available. Applications of forecasting in various sports can draw from the results and the betting odds of a few thousands of matches (Baker and McHale 2013; Forrest et al. 2005; Kovalchik 2016; Štrumbelj and Šikonja 2010; Štrumbelj and Vračar 2012). This data availability, however, is highly dependent on the sport, competition, and research question. In football, for example, data sources from European domestic leagues may include more than 30.000 matches (Angelini and Angelis 2019), while World Cups and European Championships are individual major events played with a limited number of teams and matches, whereby forecasts of these tournaments may rely on sample sizes as small as the comparison of a final ranking of 16 participating teams (Leitner et al. 2010). Internal and external factors that inherently affect the processes of any sport do have an effect on the consistency of the data through several competitions and time. Top-class sports can be subject to inconsistent rules, rule changes, or social influences beyond forecasters’ control. Examples are the fact that male tennis players play best of five sets in Grand Slam tournaments while playing best of three in ATP tour matches (see Clarke and Dyte 2000), the change from two-point rule to three-point rule in football (Riedl et al. 2015), major changes in basketball rules including a move of the three-point arc (Strumbelj et al. 2013) or possible effects of spectator exclusion due to COVID-19 measures on the home advantage (Wunderlich et al. 2021). Such effects complicate data processing and integration steps in forecasting scenarios. Further data limiting aspects are the quality (i.e., completeness and accuracy) and granularity of the available data. Artificial data helps to overcome these challenges as it enables a researcher to simulate data with a nearly unlimited size, the desired granularity as well as full consistency and quality.

A further challenge of real-world data is the heterogeneity of network structures, driven by different rules that guide each sport discipline. This set of rules may govern how the final winner is drawn from the competition, how competitors are grouped to compete or how a certain event must be executed and this structure is predefined in real-world data, The optimal model choice might depend on the competition format and its characteristics, besides all other sports-specific aspects. As an example, the same model might not be equally valuable for tennis with a knockout tournament format and football leagues with a round-robin format, or American football with a very limited number of matches per team and basketball with a very high number of matches per team. Again, artificial data enables a researcher to deliberately change competition formats while keeping all other aspects of a sport constant, which is obviously not possible in real-world applications. Thus, the isolated influence of the network structure on rating and forecasting models can be specifically analysed by means of artificial data generation.

*Step 2: The rating procedure*

Wunderlich and Memmert (2020b) outlined that the outcomes of sports events are influenced by systematic and unsystematic effects. In particular, forecasting models require modelling participant-specific systematic effects that can be denoted as quality, skill, or strength of a competitor. To account for the systematic influences of the competitors on the results, a rating procedure is the standard approach (see Barrow et al. 2013 for a definition of ratings). The rating estimation can be based on prior results (Koopman and Lit 2019) or use official ratings and rankings (Clarke and Dyte 2000; Lasek et al. 2013). While the quality of a competitor can be expressed by one single value, such as in versions of the well-established ELO rating (Glickman and Jones 1999; Hvattum and Arntzen 2010; Kovalchik 2020), it can also be beneficial to use more than one rating parameter. Dependent on the sport, this can be serve and return strengths in tennis (Newton and Aslam 2009), hazards for various types of scores in American football (Baker and McHale 2013), as well as offensive and defensive strengths in football (Koopman and Lit 2019), that can additionally be modelled as varying between home and away matches (Constantinou and Fenton 2013).

In real-world applications, it can be assumed that a large number of external and internal factors determines the strength of a competitor and its timely development. The forecaster naturally only has limited knowledge of the true underlying processes that they intend to imitate by defining a model, which is a particular challenge for ratings being an interim step of forecasting (cf. Wunderlich and Memmert 2020b). The real strength of a competitor that the rating procedure aims to estimate is not directly observable, even after the events have taken place in real-world data. Thus, the ratings quality is usually assessed indirectly by turning the rating into a forecast and then measuring its accuracy (Lasek et al. 2013). This difficulty of assessing rating quality, which has been outlined by (Wunderlich and Memmert 2020b) can be overcome by the use of artificial data. When using artificial data, the true ratings are fully known as they have been deliberately simulated. The researcher thus can directly compare estimated and true ratings without consideration of a forecasting model, as will be further elaborated on in Sect. 4.2.

*Step 3: The forecasting method*

Given the quality estimation of the teams provided by the rating procedure in Step 2, other systematic influences like the home advantage (cf. Pollard and Pollard 2005) and random processes need to be modelled to determine the probabilities for several outcomes. The forecasting method transfers the competitors’ strengths estimations (i.e. ratings) into a probabilistic forecast (i.e. a probability estimation for each possible outcome). A rather simplistic way of implementing this are Bradley-Terry models, where the outcome probabilities for a paired comparison are, in principle, solely based on the relationship between the two ratings (Cattelan et al. 2013; McHale and Morton 2011). Another straightforward possibility is using ratings as parameters of mathematical probability distributions, for example, Poisson distributions being a standard approach in football (Koopman and Lit 2015) and further goal-based sports (Karlis and Ntzoufras 2003; Marek et al. 2014). Moreover, the use of regression models to transfer ratings and potentially additional data into outcome probabilities is well established (Goddard 2005; Hvattum and Arntzen 2010). In general, sports with more than one possibility of scoring confront the forecaster with a particularly high degree of complexity that might require the use of more process-based modelling approaches. The point process model of (Baker and McHale 2013) considering several ways of scoring in American football and the Markov model of Štrumbelj and Vračar (2012), modelling the course of basketball matches by means of play-by-play data both pursue this approach. Simulation models are another viable forecasting method, particularly used for whole tournaments (Clarke and Dyte 2000; Leitner et al. 2010). However, it must be noted that this refers solely to estimating outcome probabilities and does not refer to a simulation of the whole forecasting process as discussed in this paper.

In contrast to ratings of competitors, the outcome of matches being subject of a forecasting method can be observed after the match has taken place. Still, researchers have a highly limited knowledge of the underlying processes as the observed result only represents a single instance drawn from a true probability distribution for this event. This challenge will be further elaborated on in the model validation.

*Step 4: The model validation*

As a last step, the predictive quality of the model needs to be evaluated. With regard to the two major forecasting objectives of accuracy and profitability, statistical or economic measures can be used (see Wunderlich and Memmert 2020a). Accuracy is typically focused if the purpose of the model is on forecasting methods (Kovalchik 2016) or to compare different sources of forecasts (Spann and Skiera 2009), while profitability is particularly relevant when forecasting for financial reasons (Hubáček et al. 2019) or investigating market efficiency (Angelini and Angelis 2019). In the literature, it has been widely established to report both types of measures concurrently (Baker and McHale 2013; Constantinou et al. 2012; Hvattum and Arntzen 2010; McHale and Morton 2011).

*Step 4a: Model accuracy*

The basic idea of measuring accuracy is to test how well the forecasts and the observed results fit each other. Such statistical measures include the strongly related Brier score (Cattelan et al. 2013) and rank probability score (Koopman and Lit 2019) as well as the likewise strongly related log-likelihood (Forrest et al. 2005) and ignorance score (Wheatcroft 2020). For a more detailed discussion on statistical measures and their advantages and disadvantages, refer to the work of Constantinou and Fenton (2012) and Wheatcroft (2021).

In real-world applications, the ability of a model to replicate the real processes can be assessed by extracting accuracy metrics from the observed outcomes of the events. Yet, these metrics are subject to a significant degree of randomness, as the true probability distribution for the different possible outcomes cannot be observed, even after the events have taken place. Therefore, a competition result is not necessarily reflecting the most probable outcome. Artificial data helps to exclude the noise of randomness as the forecaster has full knowledge on the probability distribution, where the actual outcome has been “drawn” from. Consequently, the true probability distribution and the estimated forecast can be directly compared.

*Step 4b: Model profitability*

The basic idea of measuring profitability is to test the model’s ability to create positive returns on the betting market. Thereafter, a new entity highly present in the sports forecasting process must be defined, the bookmakers. Bookmakers are independent entities from the sports competition with a clear profit intention. Bookmakers make use of forecasting capabilities to predict a certain event outcome and consequently post betting odds. Betting odds generally not only account for the bookmaker prediction but for the additional margin the bookmaker is expected to get out of the bets. From a forecasting practitioner point of view, knowing the estimates of the model and the betting odds offered by the bookmakers, promising bets can be identified. Using the observed results, the actual betting returns from these bets can be calculated as a measure of model profitability. Bets are only taken if they are assumed to have a positive expected value with reference to the model forecast; however, strategies can additionally differ in the choice of stakes. As a typical example for different stake selections, we refer to Hvattum and Arntzen (2010).

In real-world applications, the issue of data availability is aggravated when investigating returns of betting strategies. The initial sample size is reduced to the number of actually placed bets, which in general represents a small fraction of the total number of matches. Forrest and Simmons (2008) investigate more than 3.000 potential bets from Spanish football but report results of betting strategies based on no more than 18 to 112 bets. Similarly, McHale and Morton (2011) report results of betting strategies ranging from 54 to 123 bets, although analyzing a dataset consisting of as much as nine seasons of tennis data from ATP tournaments. Betting returns from such small numbers of bets inherently imply large noise and the danger of reporting randomly profitable betting strategies (Wunderlich and Memmert 2020a). In this sense, decent sample sizes in terms of the number of matches may still imply a highly limited informative value concerning the profitability of bets. The arbitrary sample size in artificial datasets makes it possible to circumvent this problem, reducing random noise from betting returns.

Another challenge is that profitability depends on the potential systematic inaccuracies of bookmakers in creating betting odds (Wunderlich and Memmert 2020a). In full analogy to the accuracy, the forecaster can only observe a part of the relevant information, namely the bookmaker published odds and the betting returns of a model once the event’s outcome is known. The bookmaker inaccuracies in the odds estimation or the expected values of the bets placed with regard to the true outcome probabilities of a certain event are not directly observable. In conclusion, artificial data would allow the forecaster to be in full knowledge of all processes and thus an improved and unbiased assessment of the profitability of specific models. For this reason, the artificial data framework concept presented below also comprises the bookmaker modelling. Thus, bookmakers can also be integrated in the simulation and analysis enabling improved understanding of effects of bookmaker prediction errors and profitability.

## Framework for generation and analysis of artificial sports data

**Fig. 2:**
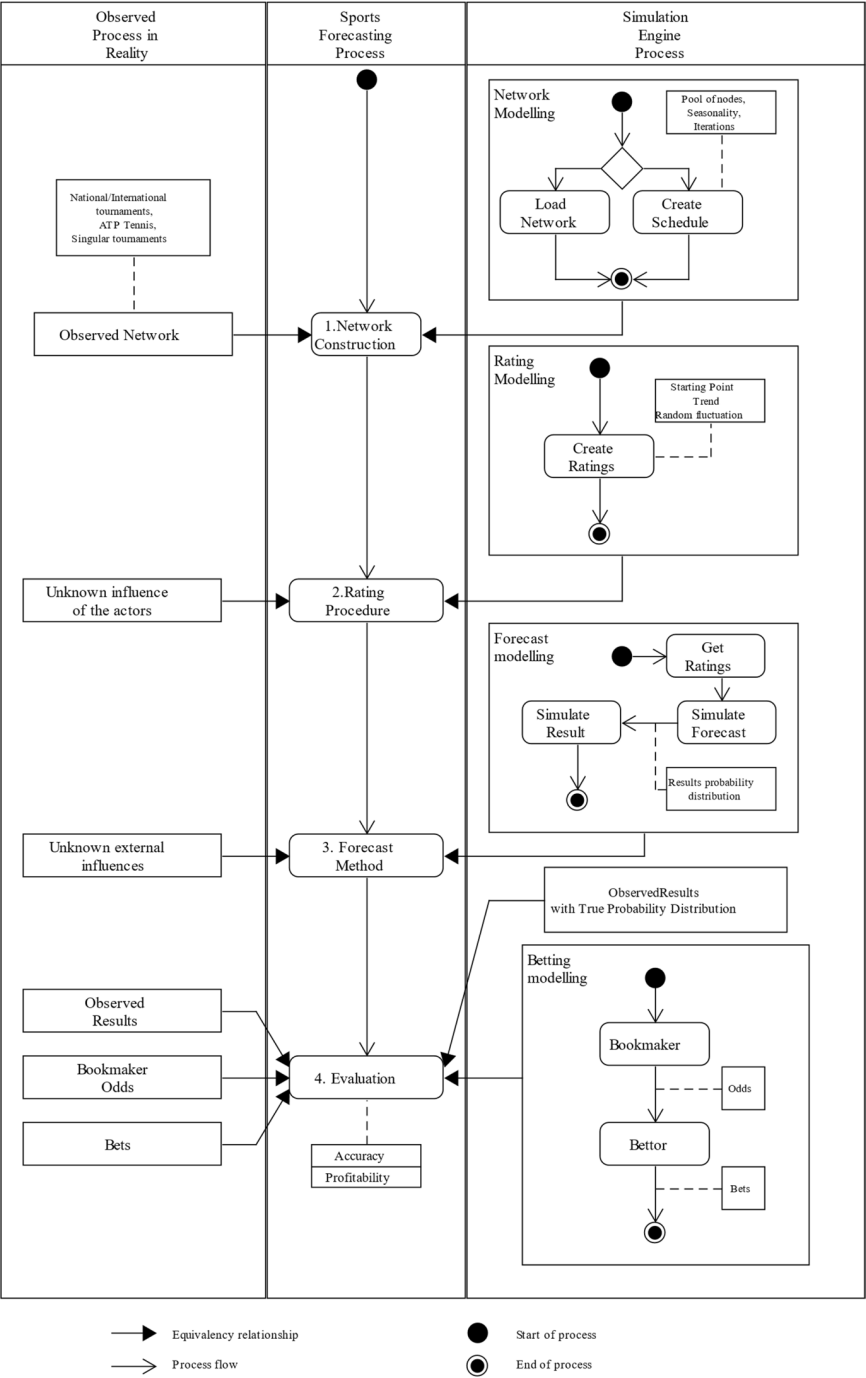
UML-based activity chart of the simulation design. The simulation engine has been designed by analyzing the observed processes in reality and the workflow of the forecasting process

The generalized process presented in Sect. 2 provides the basis to model a conceptual system to generate artificial sports data. The framework includes all the described entities present in the sports forecasting process: Competition schedules are generated and evolved in time by performing pairwise comparisons of the competitors, each competitor has attributed a certain strength that evolves in the timeline, the outcome of each comparison of competitors is drawn from a certain formula using each competitor strength among other factors. As in real-world data, estimations of the underlying processes can be added to the system: ratings can be added to the competitors to estimate their strength, forecasting methods can be implemented to predict the outcome of each comparison. At the same time, bookmakers can customize their odds, and the bettor can analyze and place bets against these odds. Every aspect of sports forecasting is included in the conceptual framework; the main difference is that the inherent nature of the data and its ground truth are fully accessible. Figure 2 provides an overview of the conceptual system design and its interaction with the model and observed process in sports forecasting. The proposed framework gains control over all the underlying unknowns and enables full customization of the process.

### Simulation engine design

The simulation engine instantiates the generalized process of sports forecasting and allows a controlled and customizable data generation and an experimental setup to properly analyze the rating procedures and forecasting methods. As presented in Fig. 2, the simulation engine gains control over the unknown and random process observed in reality and is uncertain in the sports forecasting process. Schedules are no longer external factors as they can be loaded or created by the simulation engine as networks. The simulation model enables the creation of competitions from a diverse list of sports that operate by pairwise comparisons of their competitors and different competition formats. The simulation dictates the strength of each competitor, referred to from now on as *true ratings*, and how the outcome of a pairwise comparison is going to be calculated, referred to as *true forecasts*. From now on, the conjunction between the simulated true ratings and true forecasts integrated in the competition network generation is referred as the *simulated environment* and acts as an equivalent to the real-world sports data generation. Any mathematical implementation to assign and evolve competitors’ strength can be integration on the system instantiation as well as any formula can be selected to decide on the result of each pairwise comparison for the simulation. As a result, a complete competition schedule is generated as in step 1 of the sports forecasting process. While simulating the real-world processes, the simulation engine also enables sports forecasting methods to interact with the simulated environment; thus, estimations of each competitor strength can be added as ratings and outcome predictions as forecasts.

Similarly, bookmakers and bettors are modelled. Bookmakers make use of a specific forecast to compute odds with a configured margin. In contrast, bettors propose their forecast methods and decide whether to place bets or not depending on the bookmaker odds and their own predictions. The sports forecasting steps interact with the simulation environment similarly to real-world scenarios; rating estimations do not know the true ratings, and calculated forecasts do not know the implementation governing the simulation of each confrontation. While the same metrics that can be computed with real-world data can be extracted from this setup, new insightful metrics can be combined by comparing the proposed estimation with the actual true values. The following sections describe the modelling of each simulated entity and its interfaces; while most of the models are entirely flexible and can integrate any new configuration, the simulation engine includes certain generalized implementations to provide basic examples.

#### Network modelling

Networks are defined as directed multigraphs (multidigraphs) *G* ≔ (*V*, *E*) with *V* a set of nodes and *E* a set of edges (Bang-Jensen and Gutin 2009; Newman 2010). Networks contain temporality since the edges are time-based, following the schedule of the sports competition (refer to Fig. 1). In location-based competitions such as European football leagues, source and target nodes represent the away and home teams. In other sport disciplines where the game’s location is not dependent on the nodes, the edge direction can define other attributes such as the better-ranked player or the final winner. Networks are multi directed; for instance, tennis players can face each other several times during a season. Edges of the network can contain a large variety of information related to the match itself.

**Fig. 3:**
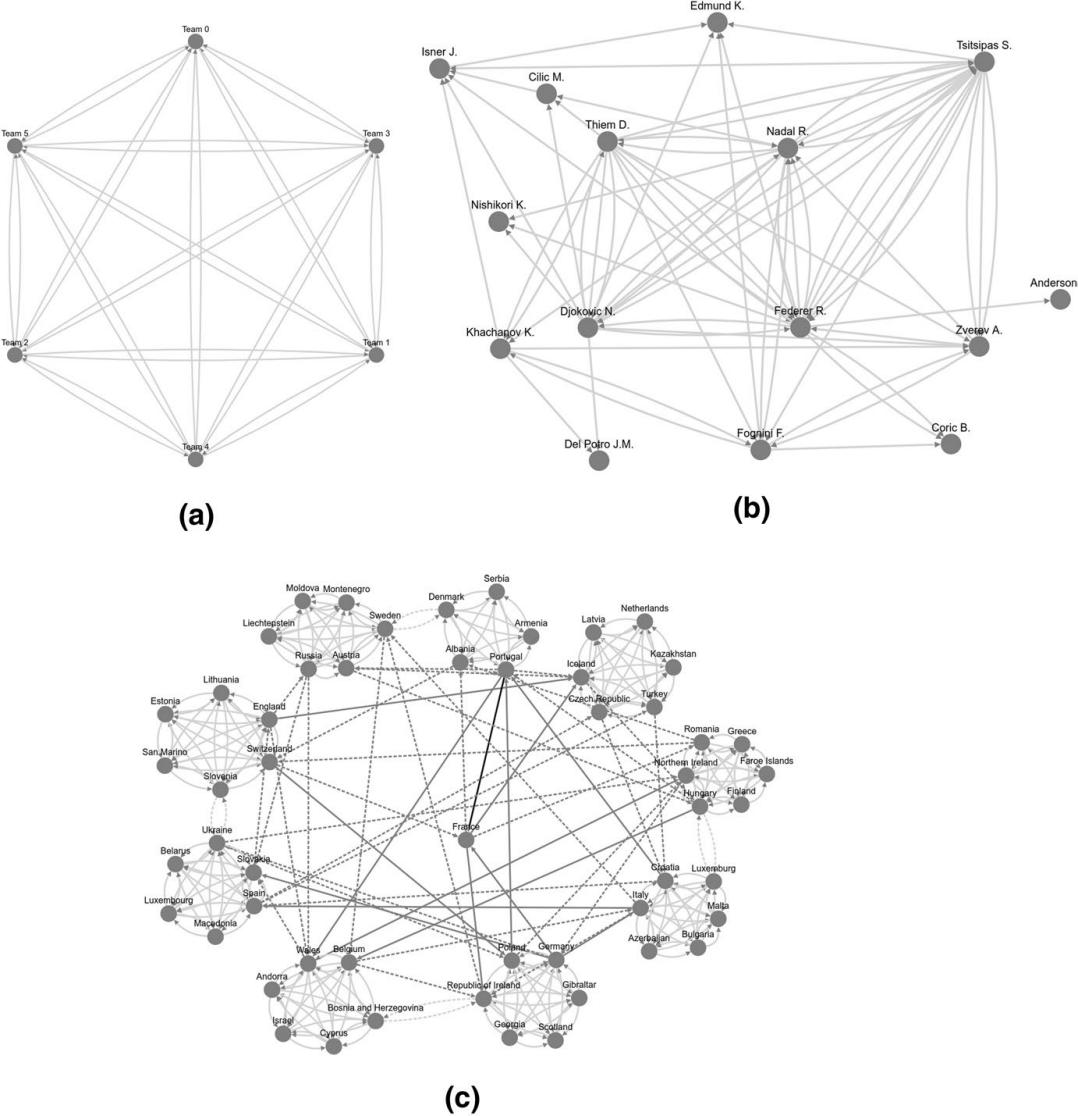
Different network topologies depending on the number of competitors, schedule, and evolution in time. A double round-robin tournament with only six teams (**a**). A real tennis ATP circuit for the top-20 tennis players (**b**). An international competition schema with different scheduling phases (**c**): competition qualifiers (light gray), qualifier play-offs (light gray dashed), competition group stage (gray dashed), knock-out final stage (gray) and, final match of the competition (black) (color figure online)

As explained in Sect. 2, different sports disciplines use different scheduling rules. The scheduling of sports competitions and regulations is represented with the topology of the simulated networks in the system. Figure 3 shows three different network topologies simulated by the system and based on real-world competitions. Figure 3a shows a simple scenario for a European Football national league with six teams. These competitions schedule the teams to face each other twice during the season in a specific order. The consequent network follows a duplicated Round-Robin tournament structure (Harary and Moser 1966). A given national league network with $\left| {V_{n} } \right|$ teams has $\left| {E_{n} } \right|$ number of games where:

$$\left| {E_{n} } \right| = \frac{{\left( {\left| {V_{n} } \right| - 1} \right)\left| {V_{n} } \right|}}{2}$$

Another typical example of a tournament is the elimination tournament. In this schedule, teams are grouped in pairs to compete. Only the winner continues playing. The modelled network forms a binary tree where only the winner nodes keep playing at each round. In this case, with a number of nodes $\left| {V_{t} } \right|$, the number of edges is $\left| {E_{t} } \right| = \left| {V_{t} } \right| - 1$. Another interesting metric of this type of tournament is the depth (D) of the tournament, or in other words, how many rounds are required to have a winner. The depth of the tournament can be computed as a function of the number of teams participating as $\left| {D_{t} } \right| = 1 + \log_{2} \left| {V_{t} } \right|$.

International competitions topologies form a composed network of simpler shapes. Figure 3c shows how a European Championship is scheduled from the qualifier stage to the final stage in two years. In other sports, such as tennis, the structure is much more complex to be represented as a system or mathematical equation. In Fig. 3b, the system simulates the shape of the 2019 ATP season for the 15 highest ranked players. Interestingly, the three topologies have properties in common and core differences, such as the variance of connection between pairs or the temporality of the network.

Networks have a certain number of iterations (number of concurrent groups of connections or rounds) and seasons (groups of iterations). Seasonality is a crucial factor in several sports disciplines. Each season is developed in a natural year in tennis, and the accumulated performances determine the players ranking. In football or basketball, lower-level teams are relegated from the league and teams from secondary divisions promoted in national leagues. The relegation and promotion mechanisms give the network a higher order than the number of teams in the league and higher diversity on edges clusters. The network’s topology is also affected by seasonality. The system design offers the possibility to create a fully functional network topology from a detailed description or mimic an existing network topology as many times as desired. Existing real-world network topologies can be loaded into the system if available.

#### Rating procedure modelling

The main goal of the rating modelling is to capture each competitor skill and the evolution in time. Thus, ratings are defined as a time-series attribute of each node in the network. The number of measurements of a node rating is not determined a priori. The simulation engine uses ratings in two different cases: A modelling of a rating can be used to represent the true rating of a competitor, meaning that the values of these ratings are going to be used as the ground truth will be the basis for the pairwise comparisons together with the true forecast methods. On the other side, ratings can also be modelled in the system to represent a calculated rating, an estimation of the true rating. Both usages of rating models follow the same abstraction in the model and are treated equally by the simulation engine.

Section 2 has already presented some examples of rating procedures in several sports. The simulation engine interacts with a standard rating interface that abstracts the implementation of any rating. A basic but robust implementation of a rating procedure can be generalized as a mathematical function yielding a numerical value at every point in time. This time series could be random or follow some trends. Following this high-level description, other ratings could be easily integrated into the model, providing a user-defined function that validates the requirements of the prerequisites of the interface. In addition to providing a broad definition of a rating procedure, the simulation engine includes an agnostic implementation of a rating procedure ruled by four different factors:

1. Rating’s starting point $S$. The initial value at the beginning of the simulation.
2. Rating’s trend $T$. A value indicating how the rating is supposed to be changing through time.
3. Rating’s trend length $T_{l} .$ The period where a trend is influencing the evolution of the rating.
4. Rating’s randomness. The magnitude of the random fluctuation to the rating concerning its trend. Two values are included in this factor, the daily random fluctuation of the rating $\Delta_{d}$ and the seasonally random fluctuation of the rating $\Delta_{s}$

The final function for a rating on a single trend length without seasonality is expressed as follows:

$$R_{i} = S + \left( { \left( {T \times i} \right) + \mathop \sum \limits_{d = 0}^{d = i} \Delta_{d } } \right)$$

where *i* isthe day number in the season and

$$S \sim P_{s}$$

$$T \sim P_{T}$$

$$\Delta_{d} \sim P_{{\Delta_{d} }}$$

and $P_{s} , P_{T} , P_{{\Delta_{d} }}$ respond to any probability distribution.

**Fig. 4:**
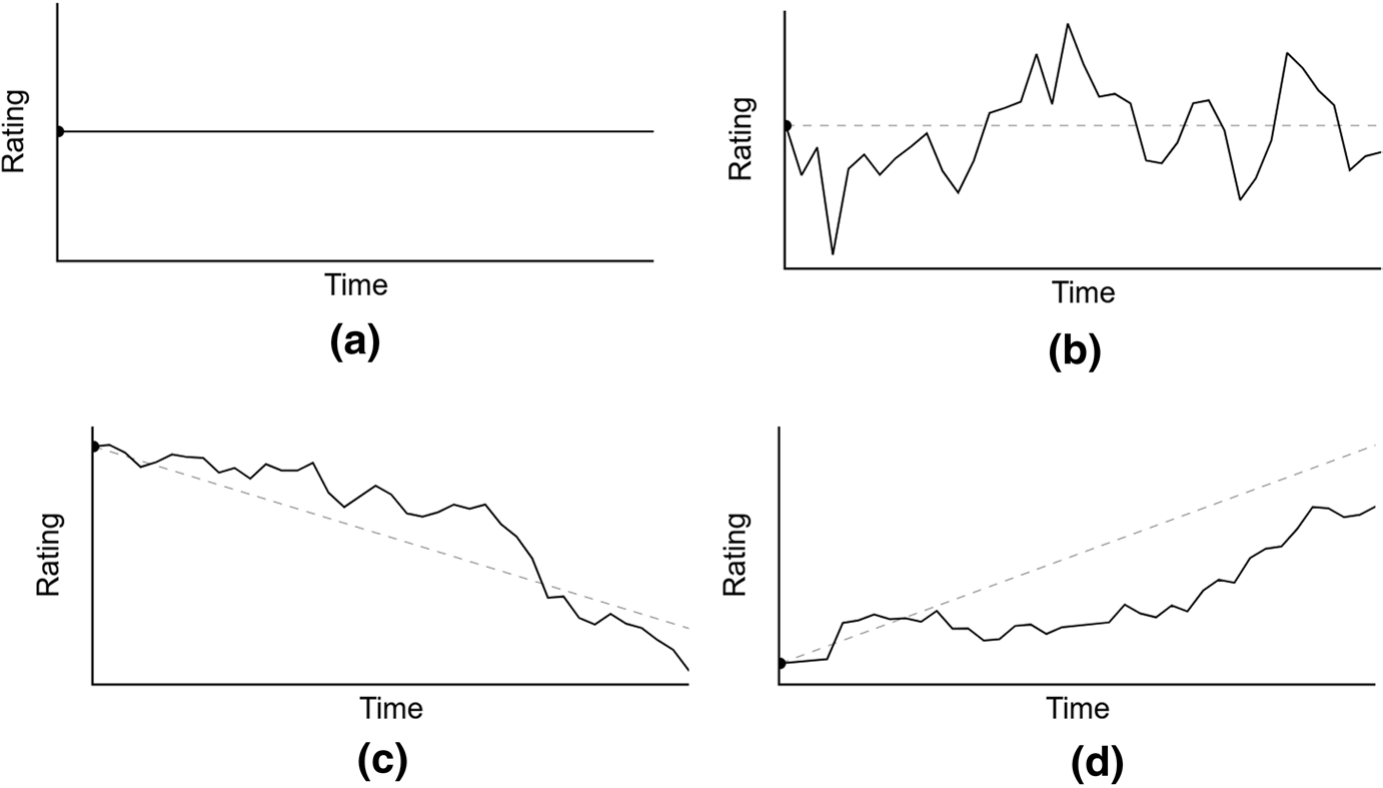
Different ratings depending on the configuration of the four factors. **a** Shows a rating with no trends and no fluctuations, **b** shows a rating with the constant trend and daily fluctuations, **c** shows a negative trend rating with daily fluctuations and **d** a positive trend with daily fluctuations

All the factors are generated independently. The trend length of the rating determines the period where a rating value follows a single function. Finally, the random seasonality fluctuation is applied at the end of each season to the rating value. Figure 4 shows four different examples of ratings generated by the system with different parameters. All four instances of rating values through time are included in the general rating procedure presented. These values could represent the strength of a tennis player during a year or the strength of a team during the second half of a basketball season. Depending on the configuration of the rating formula, the result time-series creates different behaviors, e.g., positive, stable, or negative periods, fluctuations and unexpected changes.

Although the generalized definition of the rating procedure might be helpful to generate unbiased examples, the modelling of rating procedures in the simulation enables any other assumption or implementation to be included and implemented. Thereafter, any rating procedure could be selected to represent the true ratings in the simulated environment. Specific rating procedures presented in Sect. 2 could be integrated into the simulation modelling the true ratings or the calculated ratings.

#### Forecasting method modelling

The forecasting method is configured in the system to create a forecast for each present match. The forecasting method is modelled as a processing unit that computes the predicted distribution of probabilities among the possible outcomes of an event. A forecasting model requires the match information and the competitors involved in the match. In most cases, the forecasting method also requires a previously defined rating of each for the basis of its forecasts. Like the rating procedures, the usage of a forecasting method is needed in the simulation engine to instantiate a true forecast and dictate the actual probability of each possible outcome to happen in the simulation. The simulation draws the outcome from the set of probabilities yielded by the true forecast. The forecasting methods are also present in the simulation to estimate the true forecast. The framework is designed to be able to use any forecast method for both utilities: to model the ground truth and the generation actual results and to model the calculated or proposed forecasts as long as the prerequisites of these certain methods are present in the system (i.e., certain ratings or other systematic effects). The presented modelling and definition of a forecasting method aims to include most forecasting methods present in the current state of the art, even the forecasting methods implicitly not using a rating as their basis could be included in the presented abstraction by configuring a rating with all the necessary inputs of the predictive method. The number of outcomes can be customized, e.g., two outcomes for basketball or tennis simulations or three possible outcomes (including draws) in football or chess.

#### Betting modelling

The betting modelling of the simulation engine comprises the design of bookmakers and betting actors as part of the sports forecasting process. While this modelling is not necessary to generate the core of the simulation, it implements an important part of the system where all the betting actors are simulated. The bookmaker proposes or uses a certain forecast to create the odds for each confrontation or match. The bookmaker can be configured to include systematic or random inaccuracies regarding the forecast, namely the bookmaker errors. As in reality, bookmakers are not in possession of the truth mechanism dictating the results of the events, thus their prediction is imperfect. In addition to their potential inaccuracies, the odds account for a profit margin that is intended to generate profit from the bets. Conceptually, the bookmaker entity is proposing an estimation of the true forecast as a calculated forecast. The bookmaker uses this calculated forecast and a predefined margin to generate the odds.

The system simulates a betting actor with some specific betting strategy. Conceptually, a betting strategy involves implementing a forecast for an event and the odds generated by a bookmaker for a predefined set of outcomes, therefore the bettor implements the full sports forecasting process to finally compare his predictions against the odds. Depending on the betting strategy and the bookmaker odds, the betting actor decides whether to place bets. Generally, only bets with positive expected values are placed. Betting strategies can include fixed or flexible bankrolls and unit-based or relative betting amounts (Hvattum and Arntzen 2010). The model is open to any more specific betting situations under the presented abstract definitions.

#### Evaluation module

The evaluation module is designed to allow the usage, parametrization, and analysis of any evaluation method. As discussed in Sect. 2, two main groups of forecast evaluation metrics co-exist in the sports forecasting process. The evaluation model allows the analysis to focus in either accuracy-based or profitability-based metrics. With the ability to retrieve the true probabilities from the true forecast, additional values can be calculated from these methods by substituting the actual observed results with these true probabilities. Any method present in the sports forecasting evaluation literature can be represented in the evaluation module if its requirements are fulfilled, for instance, profitable metrics are only suitable with a previous generation of bookmakers and bettors entities in the simulated environment.

## Proof of concept

This section reports how the various elements described in the artificial data framework can be integrated in a simulated sports forecasting process to gain a better understanding of forecasting scenarios. The following use cases provide some examples of how the system executes a robust and controlled environment to analyze data beyond the possibilities of real-world data. The proof of concept of the system is structured as follows: Initially, the flexible generation of data is exemplified based on the first step of the sports forecasting process. As in reality, an ad-hoc sports competition is modelled through the framework. That is, the competition schedule is decided, each competitor strength is properly simulated and the model to draw the results of each confrontation is fixed. Once the simulated environment, including true mechanisms and estimators, is generated, the focus shifts to the creation of estimators and proposed forecasts. Finally, the examples showcase how the framework improves the evaluation methods and tackles the challenges of using real-world data. Three use cases are presented, the analysis of rating procedures, the evaluation of forecast by accuracy measures and the evaluation of forecasts by profitability. The analysis and figures presented in the following sections are provided by means of an initial prototype implementation of the conceptual framework.

### Data simulation specifications

Despite the simulation not being restricted to any specific sport, the data generation of the following experiments was based on European football, and it best represents football in terms of data points, density, and evolution. The simulation consisted of ten seasons of a double round-robin tournament with eighteen competitors. No relegation or promotion procedures were specified; thus, the eighteen competitors remained competing through the ten seasons. Each competitor received a certain strength distribution by using the simulation rating presented in Sect. 3.1.2; this strength is referred by $R_{TRUE}$ through all the experiments. The rating starting point of each competitor strength was fixed to 1000. The daily trend for each competitor was drawn from a normal distribution with a mean of 0 and a standard deviation of 0.2, while the daily random fluctuation for each competitor was drawn from a normal distribution with a mean of 0 and a standard deviation of 2. Finally, a season fluctuation on each competitor strength was added to the simulation of the rating. Each specific season fluctuation for each competitor was drawn from a normal distribution with a mean of 0 and a standard deviation of 20. At every season, a new trend and a new daily fluctuation were specified for each competitor.

Once the competitor’s strength and evolution were generated, the simulation specified how each pairwise comparison would be executed. By experiment design, the possible outcomes of each comparison were specified as *home*, *draw* or *away*; however, any other combination could have also been possible. The result of each pairwise comparison scheduled is simulated by implementing an Ordered Logistic Regression (OLR) forecasting model (Arntzen and Hvattum 2020; Greene 2000). The logistic regression only uses one covariate, which sources from the difference between the two facing competitor’s ratings, and three parameters $C_{0}$, $C_{1}$ and $\beta$. The model designed to simulate the results used the true rating $R_{TRUE}$ to compute the difference between each competitor strength and $C_{0} = - 0.9$, $C_{1} = 0.3$ and $\beta = 0.006$. This OLR model, also referred as true forecast in the simulation, determined the true outcome probabilities of each comparison. The actual result of each comparison is then drawn from the respective outcome probabilities.

The explained data generation only covers the first step of the sports forecasting process. Thereafter, the second part of the data simulation tackles the generation of estimators as conceived in step 2 and 3 of the sports forecasting process. As a rating procedure candidate, an ELO Rating (Glickman and Jones 1999) was implemented and integrated with the simulation engine. The ELO rating tries to measure each competitor strength from a scoring system depending on the outcome of the matches and some initial parametrization. For testing purposes, the ELO rating added was predefined with parameters $k = 14$, $c = 10$, $d = 400$ and a home advantage of 80 points according to previous studies (Wunderlich and Memmert 2018). Equivalently, a candidate to estimate the outcome probabilities of each event was added. Despite that the conceptual system could be able to adapt a totally different forecasting method, for testing purposes, the forecasting method candidate implemented the same OLR model as the true forecast with a small but significant difference. This new forecasting method would extract the differences between each competitor from the ELO rating described above. Therefore, the differences between the true forecast and the proposed forecast would be directly related to the difference between the true ratings and the ELO estimator ratings.

This proof of concept also introduces betting actors to the simulation to showcase their functionalities. A bookmaker was configured to create odds for every comparison scheduled. Several options could be implemented with regard to the generation of the odds by the bookmaker. For testing purposes, the bookmaker accessed the true forecast of each comparison to generate the odds. However, to avoid that the bookmaker would use the exact true values, a systematic error was drawn from a standard uniform distribution for each forecast. Additionally, the bookmaker applied a 5% margin to compute the odds and add them to the simulation. To properly simulate a real-world example, none of the true processes was available to the bettor entity. In this case, the bettor would make use of the afore-mentioned proposed forecast implementing an OLR model based on the ELO Rating implementation to compute the predicted outcome probabilities. Finally, the betting strategy was configured to assign a unit to every bet placed by the bettor.

All the simulated components are summarized in Table 2. The simulated environment and the proposed estimators follow the abstraction of the sports forecasting process. In the evaluation step, only the proposed methods are subject to evaluation. While the explained data generation process is inspired by European football, similar specifications could be added to the framework to create other sports scenarios, e.g., tennis elimination tournaments or NBA playoffs. The following examples would be directly applicable to any other sports use case. Even more interestingly, unusual scenarios could have also been generated. For instance, extremely unbalanced network schedules can be the subject of analysis (i.e., competitions where some competitors are competing significantly more than others) or semantic biases could be added to any of the estimators (bookmakers with an extensive bias towards the strongest competitor, rating procedures with specific features, etc.). The following section focuses on demonstrating how the conceptual framework enables new and improved evaluation insights.

**Table 2:** Summary of the simulation specifications differencing between the simulated reality and the simulated forecasting methods

Sports forecasting process	Simulated environment	Proposed methods
Step 1: Competition Network	18 competitors in a double round-robin competition during 10 seasons	
Step 2: Rating procedure	Simulated rating $R_{TRUE}$ with $S\sim {\text{\rm N}}\left( {1000,0} \right)$ $T\sim {\text{\rm N}}\left( {0,0.2} \right)$ $\Delta_{d} \sim N\left( {0,2} \right)$ And seasonal fluctuation drawn from $N\left( {0,20} \right)$	ELO rating $R_{ELO}$ with $k = 14$, $c = 10$, $d = 400$ and a home advantage of 80 points
Step 3: Forecast method	Ordered Logistic Regression $OLR_{TRUE}$ with the covariate based on $R_{TRUE}$ and $C_{0} = - 0.9$, $C_{1} = 0.3$ and $\beta = 0.006$	Ordered Logistic Regression $OLR_{ELO}$ with covariate based on $R_{ELO}$ and $C_{0} = - 0.9$, $C_{1} = 0.3$ and $\beta = 0.006$ A forecast based on $OLR_{TRUE}$ with a ± 5% error $OLR_{BM}$ used by the bookmaker
Step 4: Evaluation	Rating accuracy Forecast accuracy Forecast profitability	

### Improved evaluation

#### Rating accuracy

The rating procedure step aims to estimate the skill or strength of a competitor. While there are many different methodologies to train and implement rating procedures, a rating evaluation is usually limited, as presented in Sect. 2. The lack of knowledge on the real strength of each competitor and how it is evolving makes it impossible to achieve a rating accuracy metric. Usually, ratings are evaluated by their predictive value, i.e., using a rating for a forecasting method and evaluating this forecast against the actual results. However, these evaluation methodologies involve other actors such as the forecast method or the randomness of the actual result that adds noise to the end goal, which evaluates the rating procedure.

In contrast to real-world applications, the conceptual system can reveal the difference between a calculated and actual rating due to the theoretical background and rating modelling. To showcase this functionality, the ELO rating $R_{ELO}$ is compared to the true rating $R_{TRUE}$.

**Fig. 5:**
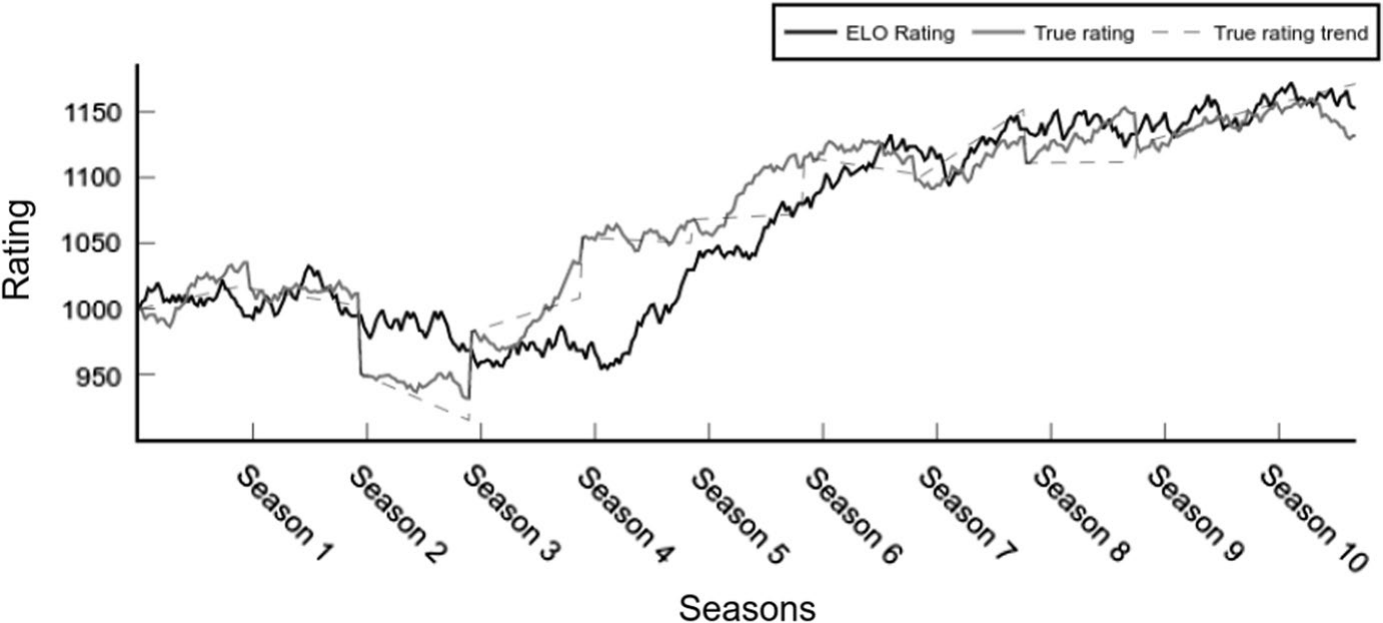
ELO Rating and True Rating evolution in time

Figure 5 plots $R_{ELO}$ in comparison to the $R_{TRUE}$ values of a single competitor. Thanks to the simulation environment, deviations and weaknesses of specific rating procedures could be identified. The test shows that the implemented $R_{ELO}$ relies upon its initialization. The initial value of $R_{ELO}$ influences how the following values will calibrate against $R_{TRUE}$ (see Fig. 5 for an example). This is usually solved by reserving part of the dataset for rating initialization. Overall, $R_{ELO}$ performs a decent estimation of the actual true rating, however, the comparison of both time series highlights some longer periods of inaccuracies. These inaccuracies are usually present in periods of high trends by $R_{TRUE}$ or proceed some seasonal fluctuations.

In the forecasting literature by means of real-world data, similar figures are shown to illustrate results or compare methods (see Fig. 1 in Koopman and Lit 2019, Figure 2 in Hvattum and Arntzen 2010 or Figures 5 and 6 in Wunderlich and Memmert 2018). However, please note that a comparison to the true ratings is not given in the literature, as it is not observable in real-world data and only gets possible employing the simulation framework.

#### Forecast accuracy

In step 3 of the generalized sports forecasting process, forecasting methods are introduced as mechanisms to predict the probability of each possible outcome of a certain event. These methods use a set of variables as inputs including the ratings of each competitor. In real-world data applications, the accuracy of these forecasts is extracted by analyzing them against the observed results. A well-established metric to measure the accuracy of forecasts expressed as probability distributions is the Rank Probability Score (RPS) (Koopman and Lit 2019). However, the RPS judges a forecast by how close the distribution is to the observed value. As discussed above, in sports forecasting as well as many other forecasting disciplines, there is a significant degree of randomness in the observed value, possibly misleading the evaluation processes.

Thanks to the simulated environment, the actual probability distributions are known, and the evaluation process can be improved. Table 3 gives an overview of the evaluation metrics available for analysis. When evaluating a proposed forecast such as the $OLR_{ELO}$, the RPS value would refer to the distance between the model probabilities and the observed values in the simulation. The RPS then contains systematic errors due to the inaccuracies of $OLR_{ELO}$ and unsystematic errors due to, for instance, randomness on the observed values. This same procedure could be used to evaluate how close the true forecast $OLR_{TRUE}$ was from the observed values, in other words, how unexpected the observed values were, this metric is referred as the *true RPS*. While the true RPS still is affected by randomness, this measure eliminates systematic errors as it considers the actual probability distributions (the truth). Similarly, the evaluation of a proposed forecast could be executed independently of the observed results. Two theoretical values can then be formulated, the *expected RPS* and the *forecastability score.* The expected RPS refers to the expected score the $OLR_{ELO}$ forecast would obtain considering all the outcome possibilities and the true probability distributions. Similarly, the forecastability score is the expected RPS score the true forecast $OLR_{TRUE}$ would obtain. Consequently, the expected RPS values get rid of the unsystematic errors that the randomness of the observed results generates while still being dependent on the systematic errors of their predictions. In contrast, the forecastability score is agnostic of any errors as it is not affected by the observed results randomness neither the inaccuracies of forecast, because it uses the actual distribution of probabilities that dictates the data generation.

**Table 3:** RPS-based advanced metrics to evaluate forecast accuracy. By means of artificial data modelling, the system is able to extract metrics not affected by systematic or unsystematic errors

$OLR_{TRUE} = \left( {h = 0.27, d = 0.28, a = 0.45} \right)$*,* $OLR_{ELO} = \left( {h = 0.37, d = 0.29, a = 0.34} \right)$*, observed result* = *h*				
Metric	Implementation	Value	Systematic errors	Unsystematic errors
RPS	RPS method on $OLR_{ELO}$	0.26	Yes	Yes
True RPS	RPS method on $OLR_{TRUE}$	0.37	No	Yes
Expected RPS	Expected RPS w.r.t.$OLR_{TRUE}$	0.23	Yes	No
Forecastability Score	Expected True RPS w.r.t. $OLR_{TRUE}$	0.22	No	No

**Fig. 6:**
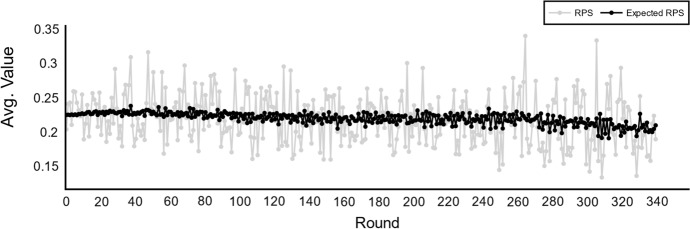
RPS versus Expected RPS average values per round. The expected RPS eliminates the effect of randomness in the measurement of accuracy of the forecasts

**Fig. 7:**
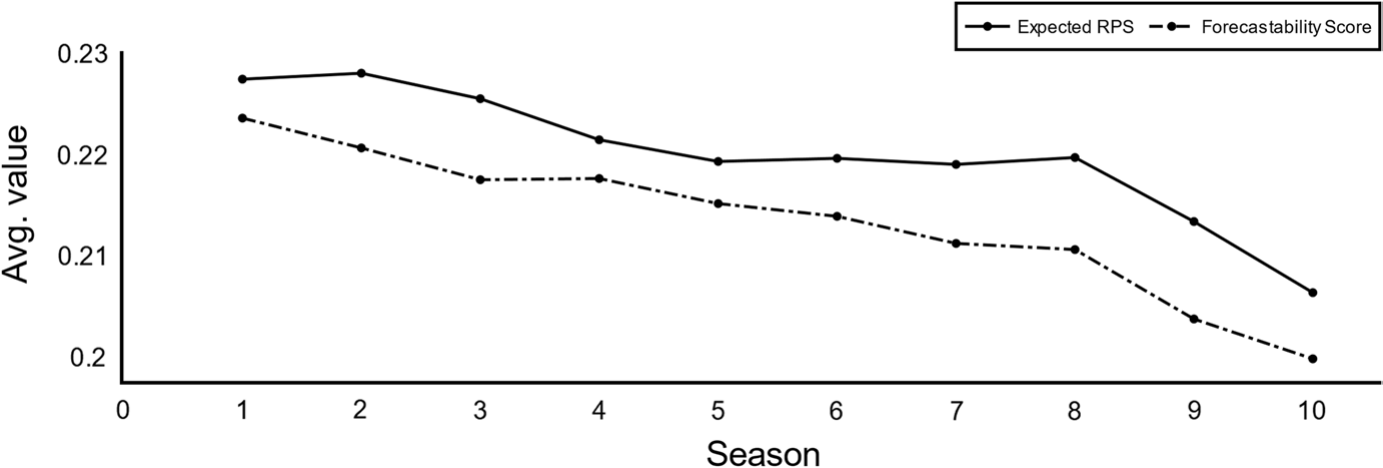
Expected RPS versus Forecastability Score average values per season simulated. The forecastability score measures the optimal forecast without systematic or unsystematic errors

Despite the fact that the metrics presented in Table 3 are theoretical, their analytical value can be useful to evaluate the accuracy of forecasting methods in practice. For instance, Fig. 6 illustrates the distribution of the RPS and the expected RPS values by competition round throughout the simulated environment. The RPS values contain a high degree of randomness while the Expected RPS is more stable. As a second example, the expected RPS values per season simulated are compared to the actual forecastability score in Fig. 7. Expected RPS versus Forecastability Score average values per season simulated. The forecastability score measures the optimal forecast without systematic or unsystematic errors.. In this case, the forecastability scores functions as an indicator of the potential accuracy that the proposed forecast could achieve, thus, a proxy for the difficulty of the problem-space. While in these brief examples only a single forecast method is used, these advanced metrics could also be of great usage to compare different methods fairly without systematic or unsystematic errors.

#### Forecast profitability

In the third case study, the aim is to show how the simulated engine provides novel tools to assess the profitability of specific betting strategies. The quality of a betting strategy is usually evaluated by calculating the achieved betting profits or losses. In a real-world example, the final profit is the only economic assessment available from a bettor’s perspective, dealing with wrong diagnoses or expectations. However, with the modelling and simulation of data, the true inherent profit expectation can be calculated from the true probabilities and reveal the differences between true and observed returns.

The betting returns were used as the main metric to evaluate the performance of the bettor. Thanks to the simulated environment, it was also possible to compute the expected betting returns. The expected returns are the measure of what a bettor could expect to get per bet on the same odds time and time again. The expected returns require the true outcome probabilities to be accessible (true forecast). Accordingly, the accumulated profit was used to compare the actual betting returns and the expected returns. The accumulated profit was defined as the sum of returns from the bettor’s bets.

**Fig. 8:**
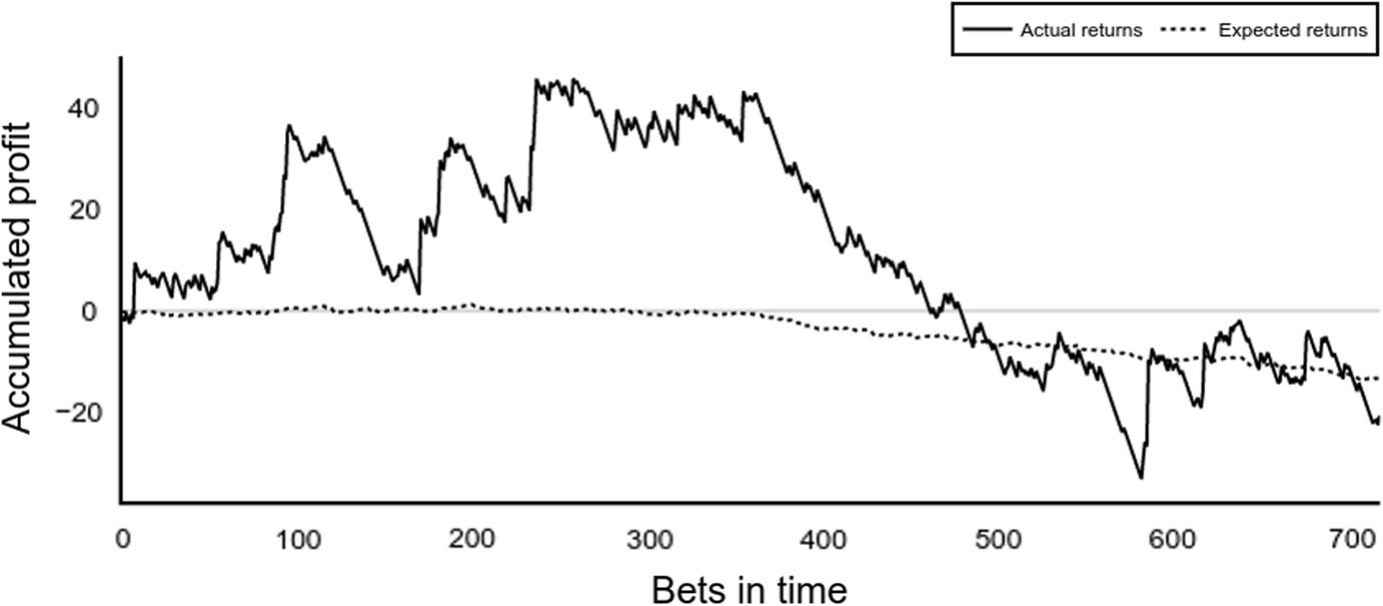
Betting returns and expected betting returns accumulated profit

Figure 8 shows the accumulated profit and the expected accumulated profit of the bets placed by the bettor in the ten seasons period. In a real-world use case, only the actual return would be visible to the bettor. The betting returns show a positive increment of profit during the first half of the betting activity. However, the plot denotes the true expected returns based on the placed bets, and the true probabilities are close to 0 and declining. The deviation from the expected returns visible in the actual returns would be a clear example where betting returns are interpreted as positive for a considerably long period without accounting for randomness in the process.

Again, similar figures reporting the evolvement of betting returns can be found in the literature (see Fig. 6 in Hubáček et al. 2019 or Figs. 5 and 6 in Constantinou and Fenton 2013). However, in analogy to the ratings, only actual returns can be found, while the comparison to expected returns only gets possible utilizing the simulation framework.

## Conclusions and applications

The sports forecasting domain has been characterized for the use of advanced mathematical and information systems theories to untangle the mechanisms of real-world scenarios. The present work contributes to this body of research from a different perspective with a focus in practical relevance and presents two enclosed utility artifacts. First, a complete abstraction of the sports forecasting process is documented and presented with a focus on demonstrating and developing a single information system for the sports forecasting domain. Second, this article introduces a comprehensive and broadly usable framework design to generate and analyze data from the domain of sports forecasting based on the presented sports forecasting process. This study has important contributions and implications in research and practice. The unifying view of the sports forecasting process is expected to contribute to the community by categorizing the different lines of research as well as the opening of new ones, as has been documented in previous design science research meta-analyses (Deng and Ji 2018). In addition, the presented process also helps practitioners and researchers to validate and contextualize their contributions by means not only of the existing literature of their peers but to the recent advances in the connected components in the process. Limitations and the challenges of the sports forecasting process by using real-world data sets have also been listed to highlight the need and benefits of a simulation framework that can model each component and accurately generate a precise simulation. The two main advantages of the framework are to enable full control over all factors in the data generation and, consequently, provide the opportunity for a more detailed data analysis. Section 4 has demonstrated these advantages by showcasing the possibility of identifying possible weaknesses in the rating procedure, extending accuracy-based metrics to account for systematic and unsystematic errors and avoiding misjudgments of betting returns by comparing actual returns to the theoretically expected returns. These analyses are impossible in applications of sports forecasting on real-world datasets. The system can integrate any other test cases or newly implemented methods than the presented in this paper and provide the same insightful functionalities. The framework presented in this work can benefit a variety of stakeholders in answering a broad range of further research questions and supporting decision-making.

First and most important, researchers can use it to better understand rating procedures and forecasting methods by testing various methods while individually varying one specific aspect of the data. Variations in network structures can help gain insights into the strengths or weaknesses of methods to handle specific competition formats (e.g., specific elimination tournaments, scheduling rules, dynamic number of competitors). Variations in the modelling of true ratings can help to reveal specific inaccuracies of rating procedures, such as inabilities to accurately account for trends, random fluctuations, or strong shifts between seasons. Likewise, forecasting methods’ variations can help identify possible systematic weaknesses of models in transferring strengths to forecasts. The final goal of these analyses would focus on using these insights to improve existing methods and re-apply them to real-world datasets. Moreover, professional gamblers can use the frameworks to understand better the connection between accuracy and profitability of forecasting models with the final ambition to create forecasting methods with improved profitability. Bookmakers, in turn, can use it to understand the impact of their inaccuracies on the risk of suffering financial losses with the ambition to incorporate this knowledge into improved odds setting procedures and risk-management tools. Similarly, sports scientists or sports organizations can use the framework to simulate the impact of changes in the structure or regulations of sports competitions. Conceivable scenarios are the adjustment of competition schedules such as the introduction of playoffs in domestic football leagues; the implementation of adjusted scoring rules such as shortened sets in tennis; attempts to improve the attractiveness of sports by stimulating a generally higher number of goals; or measures to foster equality of teams such as the introduction of salary caps. All these aspects can be specifically modelled in the context of the current framework, and the impact on ratings, probability distributions, and the evolution of competitions can be analyzed. Thus, researchers are encouraged to develop and use comparable frameworks for artificial data generation in further domains of forecasting and sports science.

While the goal of this article is to highlight the benefits of artificial data and simulation modelling in the domain of sports forecasting from a holistic view, this approach is not intended to replace studies of real data sets. In fact, it is explicitly desired and required to conceive these two approaches as complementary. While artificial data helps to overcome real-world challenges, particularly by allowing a higher level of control, new challenges are introduced, particularly as artificial data is highly dependent of the assumptions and constraints that model the simulation of a real-world problem (Koivisto 2017). To put it simply, the value of results from artificial data analysis is highly dependent on whether the processes modelled as a basis for the simulated data are actually comparable to the (unknown) real processes. Although accurately realistic artificial data and simulations are encouraged, the unknown nature of the processes involved cannot be ignored. However, the advantages of the modelling and analysis of artificial data rely beyond its validity against real-data scenarios and are centered in the increased control over all the factors and the design and study of unfeasible situations in reality. Thus, insights from artificial data could reveal answers from unconceivable questions in reality but should then always be presented in concordance with the theoretical assumptions of the simulation, discussed carefully and consequently, be validated and applied in real-world data applications.

The utility and evaluation of the presented framework has been based in the current theories and methods available in the sports forecasting domain and evaluated by their research and practice relevance. It is expected that the adaptation and instantiation of the process model and the artificial data framework would help to increase the knowledge base in sports forecasting. Similarly, the execution of rigorous research is expected to undoubtedly determine further iterations. Based on Hevner and Chatterjee (2010), the proposed artifacts must be integrated in a validation process. Relevance would ensure the practical implications of the artifacts and the innovative creation of opportunities and solutions while the rigor cycle is expected to fit the proposed artifacts with new theories, methods, and expertise from the domain of sports forecasting.

## References

[CR1] Angelini G, de Angelis L (2019). Efficiency of online football betting markets. Int J Forecast.

[CR2] Arntzen H, Hvattum LM (2020). Predicting match outcomes in association football using team ratings and player ratings. Stat Model.

[CR3] Asif M, McHale IG (2016). In-play forecasting of win probability in one-day international cricket: a dynamic logistic regression model. Int J Forecast.

[CR4] Baker RD, McHale IG (2013). Forecasting exact scores in national football league games. Int J Forecast.

[CR5] Barrow D, Drayer I, Elliott P, Gaut G, Osting B (2013). Ranking rankings: an empirical comparison of the predictive power of sports ranking methods. J Quant Anal Sports.

[CR6] Booth H (2006). Demographic forecasting: 1980 to 2005 in review. Int J Forecast.

[CR7] Cattelan M, Varin C, Firth D (2013). Dynamic Bradley-Terry modelling of sports tournaments. J Roy Stat Soc Ser C (Appl Stat).

[CR8] Clarke SR, Dyte D (2000). Using official ratings to simulate major tennis tournaments. Int Trans Operational Res.

[CR9] Constantinou AC, Fenton NE (2012). Solving the problem of inadequate scoring rules for assessing probabilistic football forecast models. J Quant Anal Sports.

[CR10] Constantinou AC, Fenton NE (2013). Determining the level of ability of football teams by dynamic ratings based on the relative discrepancies in scores between adversaries. J Quant Anal Sports.

[CR11] Constantinou AC, Fenton NE, Neil M (2012). pi-football: a Bayesian network model for forecasting association football match outcomes. Knowl-Based Syst.

[CR12] de Saá Guerra Y, Martín González JM, Sarmiento Montesdeoca S, Rodríguez Ruiz D, García-Rodríguez A, García-Manso JM (2012). A model for competitiveness level analysis in sports competitions: application to basketball. Physica A.

[CR13] Forrest D, Simmons R (2008). Sentiment in the betting market on Spanish football. Appl Econ.

[CR14] Forrest D, Goddard J, Simmons R (2005). Odds-setters as forecasters: the case of English football. Int J Forecast.

[CR15] Glickman M, Jones A (1999). Rating the chess rating system. Chance.

[CR16] Goddard J (2005). Regression models for forecasting goals and match results in association football. Int J Forecast.

[CR17] Gorr W, Olligschlaeger A, Thompson Y (2003). Short-term forecasting of crime. Int J Forecast.

[CR18] Green KC, Armstrong JS, Soon W (2009). Validity of climate change forecasting for public policy decision making. Int J Forecast.

[CR19] Greene WH (2000). Econometric analysis.

[CR20] Groll A, Heiner J, Schauberger G, Uhrmeister J (2020). Prediction of the 2019 IHF World Men’s Handball Championship – a sparse Gaussian approximation model. JSA.

[CR21] Harary F, Moser L (1966). The theory of round robin tournaments. Am Math Mon.

[CR22] Heuer A, Rubner O (2009). Fitness, chance, and myths: an objective view on soccer results. Eur Phys J B.

[CR23] Hevner A, Chatterjee S (2010). Design science research in information systems. Design research in information systems: theory and practice.

[CR24] Hevner M, Park R (2004). Design science in information systems research. MIS Q.

[CR25] Hong T, Pinson P, Fan S, Zareipour H, Troccoli A, Hyndman RJ (2016). Probabilistic energy forecasting: global energy forecasting competition 2014 and beyond. Int J Forecast.

[CR26] Horvat T, Job J (2020). The use of machine learning in sport outcome prediction: a review. Wires Data Mining Knowl Discov.

[CR27] Hubáček O, Šourek G, Železný F (2019). Exploiting sports-betting market using machine learning. Int J Forecast.

[CR28] Hvattum LM, Arntzen H (2010). Using ELO ratings for match result prediction in association football. Int J Forecast.

[CR29] Jahangirian M, Naseer A, Stergioulas L, Young T, Eldabi T, Brailsford S, Patel B, Harper P (2012). Simulation in health-care: lessons from other sectors. Oper Res Int J.

[CR30] Karlis D, Ntzoufras I (2003). Analysis of sports data by using bivariate poisson models. J Royal Statistical Soc D.

[CR31] Koivisto M (2017). Pitfalls in modeling and simulation. Procedia Computer Science.

[CR32] Koopman SJ, Lit R (2015). A dynamic bivariate poisson model for analysing and forecasting match results in the English premier league. J R Stat Soc A.

[CR33] Koopman SJ, Lit R (2019). Forecasting football match results in national league competitions using score-driven time series models. Int J Forecast.

[CR34] Kovalchik SA (2016). Searching for the GOAT of tennis win prediction. J Quant Anal Sports.

[CR35] Kovalchik S (2020). Extension of the Elo rating system to margin of victory. Int J Forecast.

[CR36] Lai M, Meo R, Schifanella R, Sulis E (2018). The role of the network of matches on predicting success in table tennis. J Sports Sci.

[CR37] Lasek J, Szlávik Z, Bhulai S (2013). The predictive power of ranking systems in association football. IJAPR.

[CR38] Leitner C, Zeileis A, Hornik K (2010). Forecasting sports tournaments by ratings of (prob)abilities: a comparison for the EURO 2008. Int J Forecast.

[CR39] Lessmann S, Sung M-C, Johnson JE (2010). Alternative methods of predicting competitive events: an application in horserace betting markets. Int J Forecast.

[CR40] Liebscher S, Kirschstein T (2017). Predicting the outcome of professional darts tournaments. Int J Perform Anal Sport.

[CR41] Lin X, Genest C, Banks DL, Molenberghs G, Scott DW, Wang J-L (2014). Past, present, and future of statistical science.

[CR42] Manner H (2016). Modeling and forecasting the outcomes of NBA basketball games. J Quant Anal Sports.

[CR43] Marek P, Šedivá B, Ťoupal T (2014). Modeling and prediction of ice hockey match results. J Quant Anal Sports.

[CR44] McHale I, Morton A (2011). A Bradley-Terry type model for forecasting tennis match results. Int J Forecast.

[CR45] McHale I, Swartz T (2019). Editorial: forecasting in sports. Int J Forecast.

[CR46] Misra A (2015). Comparative study of test data generation techniques. JITS.

[CR47] Mourtzis D, Doukas M, Bernidaki D (2014). Simulation in manufacturing: review and challenges. Procedia CIRP.

[CR48] Newton PK, Aslam K (2009). Monte Carlo tennis: a stochastic Markov chain model. J Quant Anal Sports.

[CR49] Park J, Newman MEJ (2005). A network-based ranking system for US college football. J Stat Mech Theory Exp.

[CR50] Pollard R, Pollard G (2005). Long-term trends in home advantage in professional team sports in North America and England (1876–2003). J Sports Sci.

[CR51] Riedl D, Heuer A, Strauss B (2015). Why the three-point rule failed to sufficiently reduce the number of draws in soccer: an application of prospect theory. J Sport Exerc Psychol.

[CR52] Soto Valero C (2016). Predicting win-loss outcomes in MLB regular season games – a comparative study using data mining methods. Int J Comput Sci Sport.

[CR53] Spann M, Skiera B (2009). Sports forecasting: a comparison of the forecast accuracy of prediction markets, betting odds and tipsters. J Forecast.

[CR54] Stekler HO, Sendor D, Verlander R (2010). Issues in sports forecasting. Int J Forecast.

[CR55] Štrumbelj E, Šikonja MR (2010). Online bookmakers’ odds as forecasts: the case of European soccer leagues. Int J Forecast.

[CR56] Štrumbelj E, Vračar P (2012). Simulating a basketball match with a homogeneous Markov model and forecasting the outcome. Int J Forecast.

[CR57] Strumbelj E, Vračar P, Robnik-Šikonja M, Dežman B, Erčulj F (2013). A decade of euroleague basketball: an analysis of trends and recent rule change effects. J Hum Kinet.

[CR58] Taylor JW, Buizza R (2004). A comparison of temperature density forecasts from GARCH and atmospheric models. J Forecast.

[CR59] Timmermann A (2000). Density forecasting in economics and finance. J Forecast.

[CR60] Vaughan Williams L, Stekler HO (2010). Sports forecasting. Int J Forecast.

[CR61] Venable J, Pries-Heje J, Baskerville R (2016). FEDS: a framework for evaluation in design science research. Eur J Inf Syst.

[CR62] Wheatcroft E (2020). A profitable model for predicting the over/under market in football. Int J Forecast.

[CR63] Wheatcroft E (2021). Evaluating probabilistic forecasts of football matches: the case against the ranked probability score. J Quant Anal Sports.

[CR64] Wilks DS, Wilby RL (1999). The weather generation game: a review of stochastic weather models. Prog Phys Geogr Earth Environ.

[CR65] Wolfers J, Leigh A (2002). Three tools for forecasting federal elections: lessons from 2001. Aust J Polit Sci.

[CR66] Wunderlich F, Memmert D (2018). The betting odds rating system: using soccer forecasts to forecast soccer. PLoS ONE.

[CR67] Wunderlich F, Memmert D (2020). Are betting returns a useful measure of accuracy in (sports) forecasting?. Int J Forecast.

[CR68] Wunderlich F, Memmert D (2020). Forecasting the outcomes of sports events: A review. Eur J Sport Sci.

[CR69] Wunderlich F, Weigelt M, Rein R, Memmert D (2021). How does spectator presence affect football? Home advantage remains in European top-class football matches played without spectators during the COVID-19 pandemic. PLoS ONE.

[CR70] Zhang X (2018). Application of discrete event simulation in health care: a systematic review. BMC Health Serv Res.

[CR71] Bang-Jensen J, Gutin G (2009) Digraphs: Theory, algorithms and applications / Jøorgen Bang-Jensen, Gregory Gutin, 2nd edn. Springer monographs in mathematics. Springer, London

[CR72] Deng Q, Ji S (2018) A review of design science research in information systems: concept, process, outcome, and evaluation. PAJAIS 1–36. 10.17705/1pais.10101

[CR73] Nederlandse Online Gambling Associatie (2015) Sports betting: commercial and integrity issues. https://no-ga.nl/wp-content/uploads/2020/08/Sports-Betting-Commercial-and-Integrity-Issues.pdf. Accessed 7 February 2022

[CR74] Newman MEJ (2010) Networks: an introduction/M.E.J. Newman. Oxford University Press, Oxford

